# Editorial: Bad Bugs in the XXIst Century: Resistance Mediated by Multi-Drug Efflux Pumps in Gram-Negative Bacteria

**DOI:** 10.3389/fmicb.2016.00833

**Published:** 2016-05-31

**Authors:** Attilio V. Vargiu, Klaas M. Pos, Keith Poole, Hiroshi Nikaido

**Affiliations:** ^1^Department of Physics, University of CagliariMonserrato, Italy; ^2^Institute of Biochemistry, Goethe-UniversityFrankfurt am Main, Germany; ^3^Department of Biomedical and Molecular Sciences, Queen's UniversityKingston, ON, Canada; ^4^Department of Molecular and Cell Biology, University of California, BerkeleyBerkeley, CA, USA

**Keywords:** antibiotic resistance, multi-drug-resistant pathogens, efflux pumps, superbugs, bacterial resistance mechanisms

The discovery of antibiotics represented a key milestone in the history of medicine. However, with the rise of these life-saving drugs came the awareness that bacteria deploy defense mechanisms to resist these antibiotics, and they are good at it. Today, we appear at a crossroads between discovery of new potent drugs and omni-resistant superbugs. Moreover, the misuse of antibiotics in different industries has increased the rate of resistance development by providing permanent selective pressure and, subsequently, enrichment of multidrug resistant pathogens. As a result, antimicrobial resistance has now become an urgent threat to public health worldwide (http://www.who.int/drugresistance/documents/surveillancereport/en/). The development of multidrug resistance (MDR) in an increasing number of pathogens, including *Pseudomonas, Acinetobacter, Klebsiella, Salmonella, Burkholderia*, and other Gram-negative bacteria is a serious issue. Membrane efflux pump complexes of the Resistance-Nodulation-Division (RND) superfamily play a key role in the development of MDR in these bacteria. These pumps, together with other transporters, contribute to intrinsic and acquired resistance of bacteria toward most, if not all, of the compounds available in our antimicrobial arsenal. Given the enormous drug polyspecificity of MDR efflux pumps, studies on their mechanism of action are extremely challenging, and this has negatively impacted both on the development of new antibiotics that are able to evade these efflux pumps and on the design of pump inhibitors. The collection of articles in this eBook, published as a Research Topic in Frontiers in Microbiology, section of Antimicrobials, Resistance, and Chemotherapy, aims to update the reader about the latest advances on the structure and function of RND efflux transporters, their roles in the overall multidrug resistance phenotype of Gram-negative pathogens, and on the strategies to inhibit their activities.

RND transporters reside in the inner membrane, and in Gram-negative bacteria they form trans-envelope complexes together with periplasmic membrane fusion proteins (MFPs) and outer membrane factors (OMFs). As tripartite entities, these macromolecular complexes are powerful machines that expel multiple antibiotics across outer membranes. In recent years, much of the research efforts have been focused on the structures of the RND, MFP, and the OMF components. The assembly and disassembly of the tripartite efflux pump components is so crucial to their physiological role in resistance, that much research effort has been directed toward both the elucidation of the structures and functional dynamics of individual components and the *in vivo* and *in vitro* assembly and function of the tripartite systems. X-ray crystallography has provided an understanding of the molecular basis of drug transport by RND-type efflux pumps. Yamaguchi et al. stress critical points related to structure and function of these tripartite pumps, including the lack of consensus on the number of adaptor proteins and the molecular basis for multidrug recognition by two voluminous promiscuous binding pockets in the RND transporter, and they formulate a multisite-drug-oscillation hypothesis to describe their activity. Substrate-mediated conformational changes in RND efflux pumps are also investigated *in silico* by Wang et al. The authors use molecular dynamics simulations of AcrB in complex with substrates to discover an intrinsic allostery in the transport mechanism upon binding of a second substrate. Dreier and Ruggerone analyze how subtle differences in the physicochemical features of antibiotics belonging to the same family determine their diverse susceptibility to efflux mechanisms. They highlight the importance of combining different high-resolution techniques to gain insights on substrate recognition patterns. Broutin and co-workers (Monlezun et al.) focus on the outer membrane channel OprM of *Pseudomonas aeruginosa*, investigating the presence of N-terminal modifications. They present a new X-ray structure solved in a new space group, making it possible to model the N-terminal residue of OprM as a palmitoylated cysteine.

Several factors are critical for the proper function of the tripartite system: drug binding, interaction with MFPs and OMFs, proton relay through the transmembrane domain, and trimerization of the transporter. Regarding the latter process, Wang et al. use AcrAB-TolC of *Escherichia coli* as model to explore the mechanism of function recovery of the AcrB_P223G_ variant, which compromises AcrB trimerization and drastically reduces the drug efflux activity. Their study highlights how modulation of several critical factors can lead to the proper functioning of the pump. Zgurskaya et al. further analyze how the reaction cycles of transporters are coupled to the assembly of the trans-envelope complexes. Using a combination of biochemical, genetic and biophysical approaches, the authors reconstruct the sequence of events leading to the assembly of transenvelope drug efflux complexes, and characterize the roles of periplasmic and outer membrane proteins in this process. In particular, they propose that OMF recruitment is triggered by binding of effectors (substrates) to MFP or MFP-RND complexes. Bavro and co-workers investigate in detail the architecture and roles of periplasmic adaptor proteins in tripartite efflux pump assemblies (Symmons et al.). They stress how recognition between the MFPs and OMFs is essential for pump assembly and function, and how targeting this interaction may provide a novel avenue for combating RND pump-mediated multidrug resistance. Picard and co-workers design a new test permitting investigation of the assembly of the MexAB-OprM efflux system of *P. aeruginosa* with only tens of microgram of protein (Ntsogo Enguene et al.). The method relies on the streptavidin-mediated pull-down of OprM proteoliposomes upon interaction with MexAB proteoliposomes containing biotinylated lipids. Their study gives clear evidence for the importance of MexA in promoting and stabilizing the assembly of the MexAB-OprM complex and the role of the proton motive force on the assembly and disassembly of the efflux pump.

Little of the *in vitro* work makes sense without an understanding of the occurrence, regulation, and interdependence of the efflux pumps in a physiological setting. Martins and co-workers summarize the current knowledge on RND efflux mechanisms in *E. coli*, a bacterium responsible for community and hospital-acquired infections, as well as foodborne outbreaks worldwide (Anes et al.). They review the knowledge on Acriflavine (Acr), Multidrug Transport (Mdt), and Copper transporting (Cus) efflux systems. Chen and collaborators use clinical *Salmonella typhimurium* isolates to demonstrate how acquisition of the plasmid-mediated quinolone resistance (PMQR) genes *oqxAB* and *aac(6*′*)Ib-cr* accelerates the development of fluoroquinolone resistance in this bacterium (Wong et al.). Their analysis reveals that *oqxAB* and *aac(6*′*)Ib-cr* are encoded on plasmids of various sizes and mediate resistance to ciprofloxacin, ultimately facilitating the selection of ciprofloxacin-resistant *S. typhimurium*. In their Perspective, Paulsen and collaborators summarize the current knowledge on the *Acinetobacter* chlorhexidine efflux (AceI) pump, a prototype for a novel family of multidrug efflux pumps conserved in many proteobacterial lineages (Hassan et al.). The discovery of this family raises the possibility that additional undiscovered intrinsic resistance proteins may be encoded in the core genomes of pathogenic bacteria.

Among the mechanisms that are not fully understood is the genetic regulation of efflux pumps. Morita and co-workers identify a novel MexS variant involved in up-regulation of the *mexEF-oprN* multidrug efflux operon in a clinical isolate of *P. aeruginosa* displaying multi-drug efflux-mediated resistance to fluoroquinolones, aminoglycosides, and most β-lactams. Their study constitutes the first genetic evidence that a MexS variant causes *mexEF-oprN* upregulation in *P. aeruginosa* clinical isolates. If the road toward understanding of efflux-mediated multi-drug resistance in *E. coli, Acinetobacter baumannii* and *P. aeruginosa* is rough, the characterization of efflux pumps in many other prominent pathogens, e.g., the members of the genus *Burkholderia*, lags even further behind. As in other non-enteric Gram-negatives, efflux pumps of the resistance nodulation cell division (RND) family are the clinically the most significant efflux systems in this genus. Schweizer and co-workers provide an excellent review of the current knowledge about efflux mechanisms in several *Burkholderia* species (Podnecky et al.).

Last but not least, inhibition of efflux pumps is one of the current strategies being pursued by several groups attempting to reduce the impact of efflux on antimicrobial resistance. Efflux pump inhibitors (EPIs) could be used as adjunctive therapies that would increase the potency of existing antibiotics and decrease the emergence of multidrug-resistant bacteria. In their review, Venter et al. describe how available biochemical and structural information can be translated into the discovery and development of new compounds that could reverse antimicrobial resistance in Gram-negative pathogens. Opperman and Nguyen analyze in detail the reasons why no compounds have yet progressed into clinical use. One of the major hurdles in the development of EPIs has been the lack of biochemical, computational, and structural methods that could be used to guide rational drug design. The authors review recent reports that have advanced our understanding of the mechanisms of action of several potent EPIs active against RND-type pumps.

In summary, the articles in this research topic serve as a background to the search for a deeper understanding of the mechanisms by which RND efflux pumps limit the concentration of antimicrobial compounds inside the bacterial cell. This knowledge may in turn pave the way for new, more directed inhibitor and antibiotic design to ultimately overcome antimicrobial resistance in Gram-negative pathogens.

## Author contributions

All authors listed, have made substantial, direct and intellectual contribution to the work, and approved it for publication.

## Funding

The research of AVV and KMP was conducted as part of the Translocation Consortium (www.translocation.eu) and has received support from the Innovative Medicines Initiative Joint Undertaking under Grant 115525, resources that are composed of financial contributions from the European Union's Seventh Framework Programme (FP7/2007-2013) and European Federation of Pharmaceutical Industries and Associations (EFPIA) companies. Work in the lab of KP on multidrug efflux is funded by the Canadian Institutes of Health Research and Cystic Fibrosis Canada.

### Conflict of interest statement

The authors declare that the research was conducted in the absence of any commercial or financial relationships that could be construed as a potential conflict of interest.

